# Increased risk of bladder cancer recurrence due to bacillus Calmette-Guérin shortage in Brazil

**DOI:** 10.1590/1806-9282.20231116

**Published:** 2024-05-17

**Authors:** Claudio Bovolenta Murta, Kayann Kaled Reda El Hayek, Bruno Cesar Dias, Marco Aurélio Watanabe Yorioka, Valter DellAcqua Cassao, Joaquim Francisco de Almeida Claro

**Affiliations:** 1 Instituto do Câncer do Estado de São Paulo, Department of Urology – São Paulo (SP), Brazil.; 2 Hospital Brigadeiro, Men’s Health Centre, Division of Urology – São Paulo (SP), Brazil.

**Keywords:** Non-muscle invasive bladder neoplasms, Transurethral resection of bladder, BCG vaccine, Recurrence, Disease progression

## Abstract

**OBJECTIVE:**

Our study aimed to evaluate the impact of bacillus Calmette-Guérin shortage on recurrence and progression in patients with non-muscle invasive bladder cancer in a Brazilian cohort.

**METHODS:**

We retrospectively reviewed the clinicopathological data of 409 patients who had their first transurethral resection of the bladder tumor for intermediate or high-risk non-muscle invasive bladder cancer between June 2014 and May 2021 in a tertiary public hospital in Brazil. Patients included had non-muscle-invasive urothelial carcinoma of the bladder resected completely for the first time, regardless of bacillus Calmette-Guérin use. Low-risk disease patients were excluded from the analysis. Demographic, clinicopathological, and bacillus Calmette-Guérin use data were collected from our database. Recurrence and progression data were obtained from patient records or through telephone interviews. Recurrence-free survival and progression-free survival were calculated from the date of transurethral resection of the bladder tumor until the events of recurrence, progression, last office visit, or phone interview.

**RESULTS:**

Within a median follow-up period of 26.7 months, 168 (41.1%) patients experienced a recurrence in a median time of 27 months (95%CI 16.1–38). Bacillus Calmette-Guérin was administered to 57 (13.9%) individuals after transurethral resection of the bladder tumor. Patients with ≥3 lesions (p<0.001), those with lesions >3 cm (p=0.02), and those without bacillus Calmette-Guérin treatment (p<0.001) had shorter recurrence-free survival. According to a Cox multivariate regression model, bacillus Calmette-Guérin use was independently associated with a reduced recurrence rate, with an HR of 0.43 (95%CI 0.25–0.72). Out of the patients studied, 26 (6.4%) experienced progression. T1 stage (p<0.001) and high-grade (p<0.001) were associated with shorter progression-free survival. Bacillus Calmette-Guérin did not influence bladder cancer progression. In the Cox multivariate analysis, high-risk disease was independently associated with progression (p<0.001).

**CONCLUSION:**

Our study confirms that non-muscle invasive bladder cancer exhibits a high recurrence rate. The use of adjuvant bacillus Calmette-Guérin in intermediate and high-risk patients significantly reduces this rate. Furthermore, the bacillus Calmette-Guérin shortage could have negatively impacted these patients.

## INTRODUCTION

Bladder cancer (BC) is a common urological neoplasm, accounting for more than 10,000 new cases in Brazil in 2020^1^. Fortunately, approximately 75% of patients will present with non-muscle-invasive bladder cancer (NMIBC)^2^, which is primarily treated with transurethral resection of the bladder tumor (TURBT)^3^.

Although the surgical treatment is minimally invasive and highly effective, up to 78% of patients will experience recurrence, and 40% will progress to muscle-invasive bladder cancer (MIBC) within 5 years^4,5^. To stratify the patients accordingly, the European Association of Urology proposed a new classification of patients based on their risk of progression in 2021^3^. Patients with intermediate, high, and even very high risk of progression should receive intravesical immunotherapy with bacillus Calmette-Guérin (BCG) because it reduces the risk of recurrence and progression when compared with TURBT alone or in combination with intravesical chemotherapy^6-8^. However, patients have suffered from BCG shortages in recent years worldwide, impacting not only recurrence and progression rates but on a wider scale also significant economic impact^9,10^.

To assess the impact of the BCG shortage on clinical outcomes in a Brazilian cohort of patients with NMIBC, we evaluated patients submitted to their first TURBT in a tertiary public hospital. This evaluation focused on recurrence and progression in relation to the use of adjuvant BCG.

## METHODS

**Figure 1:**
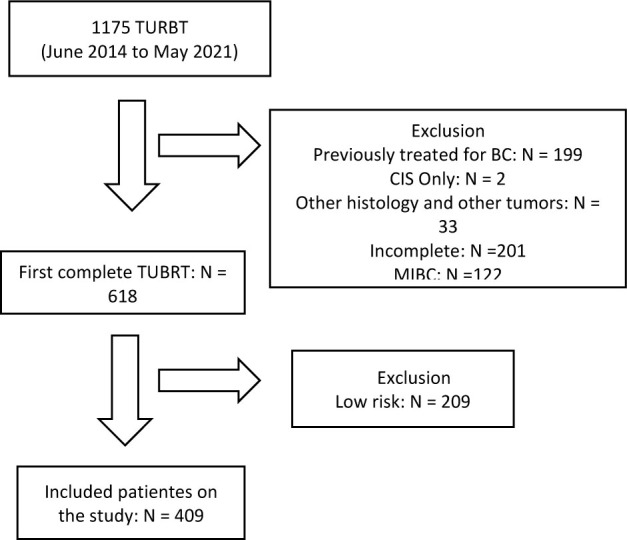
Flowchart of included patients.

Patients who underwent their first complete TURBT for papillary NMIBC at our institution from June 2014 to May 2021 were selected for analysis. They were followed up, and data were collected until December 2021. The exclusion criteria were CIS only with no visible papillary tumor, primary histologies other than urothelial carcinoma (although other subtypes were allowed as long as the urothelial type was the most common in the specimen), incomplete resections, patients with previous treatment for BC or other neoplasms, and MIBC at first TURBT. According to EAU risk groups^5^, only patients with intermediate or high-risk diseases were included in the final analysis, regardless of adjuvant BCG use. The flowchart is demonstrated in Figure 1.

Data were retrospectively collected and retrieved following approval by the local ethical committee (approval #5.194.005, date of approval: January 06, 2022). All patients provided informed consent to participate in this study. The collected data were age at surgery, sex, smoking history, presence of hydronephrosis in the pre-operative imaging, data of the first TURBT such as the number of lesions, size of the most extensive lesion, presence of muscular propria, and carcinoma in situ (CIS), final pathology grade according to WHO 2016 and stage, and BCG use. Information on recurrence, progression, metastasis, or death was obtained from patient records or through telephone interviews.

Recurrence was defined when any of the following events occurred: diagnosis of NMIBC or MIBC, metastasis, or death from BC. Progression was defined as MIBC diagnosed on a TURBT, metastasis, or death from BC. Low-risk patients were followed with cystoscopy 3 months after the resection and yearly thereafter, having had ultrasound exams performed in between. Patients with T1 disease and high grade without the presence of muscular propria were submitted to re-TUR in 4–6 weeks. Intermediate and high-risk patients were followed up with cystoscopy every 6 months and with an ultrasound and cytology yearly. When available, intravesical BCG instillations were offered to all patients with intermediate and high-risk patients according to EAU guidelines on NMIBC^3^.

Parametric data are presented as mean and standard deviation (SD) or median and interquartile range (IQR) for those with normal or non-normal distribution, respectively. Recurrence-free survival (RFS) was calculated from the date of TURBT until first local or distant recurrence, and progression-free survival (PFS) was calculated until progression, metastasis, or death event. The data were collected from records from the last office visit, or through telephone interviews. The log-rank test was used to correlate the clinicopathological variables and BCG use with RFS and PFS using the SPSS v.28.0. A p<0.05 was considered statistically significant. To identify independent factors associated with recurrence and progression, a Cox regression model was used to estimate the hazard ratio (HR) and the 95% confidence interval (CI) values. All significant variables were tested in a univariate Cox regression model, and those which showed to be associated with the studied outcome were included in the multivariate model to confirm their independent association.

## RESULTS

From June 2014 to May 2021, 1175 patients underwent TURBT at our institution. Of these, 618 patients underwent their first complete resection for BC with papillary tumors, and 409 of these, who met the criteria for BCG treatment, were included in the final analysis. The median age at surgery was 69.4 years (IQR 14.9), and the median follow-up period was 26.7 months (IQR 25.7), as shown in Table 1. Most were men and current or former smokers (Table 1). Only 26 (6.4%) patients presented hydronephrosis on pre-procedural imaging.

**Table 1:** Clinicopathological characteristics of the patients submitted to transurethral resection of the bladder tumor for non-muscle invasive bladder cancer (n=409).

Follow-up in months—median (IQR)	26.7 (25.7)
Age at surgery in years—median (IQR)	69.4 (14.9)
Men (%)	317 (77.5)
Smoking history (%)	300 (73.3)
Hydronephrosis (%)	26 (6.4)
Mean number of lesions (IQR)	1.0 (2.0)
1–3 (%)	331 (81.9)
>3 (%)	73 (18.1)
Mean size of the biggest lesion (IQR)	4.0 (2.0)
<3 cm (%)	84 (20.5)
≥3 cm (%)	327 (79.5)
Grade	
Low (%)	162 (39.6)
High (%)	247 (60.4)
Stage	
Ta (%)	296 (72.4)
T1 (%)	113 (27.6)
BCG	
No (%)	352 (86.1)
Induction (%)	45 (11.0)
Induction + maintenance (%)	12 (2.9)
Group risk (EAU)	
Intermediate	241 (58.9)
High/very high	168 (41.1)

TURBT: transurethral resection of bladder cancer; NMIBC: non-muscle invasive bladder cancer; n: number of patients; IQR: interquartile; BCG: bacillus Calmette-Guérin; EAU: European Association of Urology.

Intraoperative findings during TURBT and the pathological results are summarized in Table 1. According to the new EAU risk classification for NMIBC, 241 patients (58.9%) were classified as intermediate risk and 168 (41.1%) as high or very high risk of progression. The clinical and pathological characteristics of these patients are detailed in Table 1.

Bacillus Calmette-Guérin usage was low across the cohort, with only 13.9% of patients receiving at least one cycle of induction of BCG. Only 2.9% of patients received BCG maintenance therapy (Table 1).

**Figure 2:**
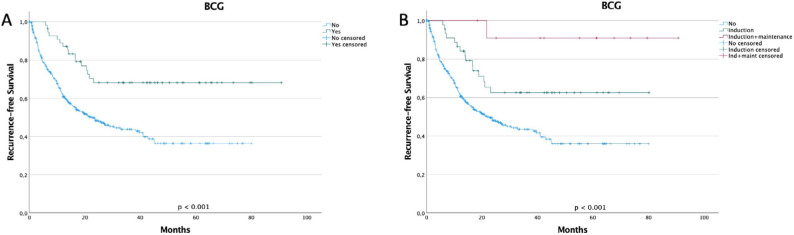
Kaplan-Meier curve for recurrence-free survival according to the use of bacillus Calmette-Guérin for patients submitted to transurethral resection of intermediate and high-risk non-muscle invasive bladder cancer (n=409). (A) shows the patients stratified for use or no use of bacillus Calmette-Guérin. In (B), patients were stratified into patients who have not received bacillus Calmette-Guérin, those who received only induction, and those who received induction plus maintenance. Bacillus Calmette-Guérin use had the longest RFS, especially if maintenance was used (log-rank test; p<0.001).

Recurrence was observed in 188 patients (46.0%) during follow-up, with a median time to recurrence of 27.3 months. Gender, hydronephrosis, stage, grade, and presence of detrusor muscle, or CIS, did not correlate with RFS. Patients with more than three lesions, those with lesions larger than 3 cm, and those who did not receive BCG treatment exhibited significantly shorter RFS. When stratified by no use, induction, and induction plus maintenance, the patients who received BCG maintenance had the longest RFS (Figure 2). A univariate Cox regression analysis showed that having more than three lesions, lesions with ≥3 cm, and non-use of BCG were associated with recurrence. Further analysis with a multivariate Cox regression model controlling for these variables indicated that patients with more than three lesions or those who did not receive BCG therapy had a higher risk of BC recurrence independently (Table 2). BCG significantly reduced the recurrence rate by 57% (p=0.001).

**Table 2:** Cox regression univariate and multivariate analysis of recurrence, and progression rate of the patients submitted to transurethral resection of the bladder tumor for non-muscle invasive bladder cancer (n=409).

Recurrence risk	Univariate analysis		Multivariate analysis	
	HR (95%CI)	p	HR (95%CI)	p
Gender (male vs. female)	1.23 (0.88–1.71)	0.229		
Smoking history (yes vs. no)	1.12 (0.78–1.60)	0.538		
Hydronephrosis (yes vs. no)	1.25 (0.74–2.12)	0.410		
Number of lesions (>3 vs. 1–3)	1.78 (1.27–2.48)	<0.001	1.80 (1.29–2.52)*	<0.001
Size of lesions (≥3 vs. <3 cm)	1.58 (1.07–2.32)	0.021	1.37 (0.93–2.04)*	0.115
Grade (high vs. low)	1.03 (0.77–1.37)	0.854		
Stage (T1 vs. Ta)	0.84 (0.60–1.17)	0.301		
BCG (yes vs. no)	0.39 (0.23–0.65)	<0.001	0.43 (0.25–0.72)*	0.001
EAU risk group (high vs. intermediate)	1.14 (0.85–1.53)	0.372		
**Progression risk**	**HR (95%CI)**	**p**	**HR (95%CI)**	**p**
Gender (male vs. female)	1.05 (0.42–2.61)	0.922		
Smoking history (yes vs. no)	0.44 (0.20–0.99)	0.046	0.48 (0.21–1.07)**	0.072
Hydronephrosis (yes vs. no)	0.53 (0.07–3.95)	0.540		
Number of lesions (>3 vs. 1–3)	1.75 (0.73–4.21)	0.207		
Grade (high vs. low)	5.74 (1.72–19.14)	0.004	3.41 (0.96–12.10)**	0.058
Stage (T1 vs. Ta)	4.58 (2.08–10.09)	<0.001	2.83 (1.22–6.54)**	0.015
BCG (yes vs. no)	0.57 (0.17–1.91)	0.363		
EAU risk group (high vs. intermediate)	5.74 (2.31–14.32)	<0.001	5.35 (2.13–13.39)***	<0.001

TURBT: transurethral resection of bladder cancer; NMIBC: non-muscle invasive bladder cancer; HR: hazard ratio; CI: confidence interval; BCG: bacillus Calmette-Guérin; EAU: European Association of Urology.

*Controlled for the number of lesions, lesion size, and BCG use.

**Controlled for smoking history, grade, and stage.

***Controlled for EAU risk group and smoking history. Statistically significant values are indicated in bold.

A total of 26 (6.4%) patients progressed during the follow-up period, with a mean time of 83.7 months and a median time not reached. PFS was not influenced by gender, hydronephrosis, the number or size of the lesions, the presence of detrusor muscle, or CIS at TUR. BCG use also had no influence on PFS. Notably, those who never smoked had a shorter PFS compared with patients who were current or former smokers (p=0.040). High grade (p=0.001) and T1 stage (p<0.001) were also associated with shorter PFS. When grouped by the 2022 EAU risk classification, patients in the high-risk group had a significantly shorter PFS than those in the intermediate risk (p<0.001). The univariate Cox analysis confirmed these characteristics as associated with PFS. In the multivariate Cox regression analysis, controlled for smoking status, grade, and T stage, only the T1 stage maintained an independently significant association with progression, as shown in Table 2. The T1 stage increased the progression by a factor of 2.83 (p=0.015; Table 2). When the patients were grouped according to EAU risk groups, both high-risk classification and smoking history retained their independent association with progression (Table 2).

## DISCUSSION

In our study, we report the outcomes of recurrence and progression in a large cohort of patients with intermediate or high-risk papillary NMIBC and the potential impact that BCG shortage on these results, particularly in terms of recurrence. We found that adhering to guidelines for BCG usage could reduce recurrence rates by 60%. Throughout the study period, Brazil has experienced several BCG shortages due to production issues with its sole manufacturer^10^. Regrettably, our patients were unable to import BCG due to prohibitive costs. Furthermore, our hospital did not offer alternatives such as gemcitabine and other intravesical chemotherapies. It is noteworthy that although all patients with high-risk diseases were advised to undergo radical cystectomy, only a small proportion opted for this procedure.

Intravesical BCG therapy is a well-established treatment for NMIBC, particularly recommended for patients with an intermediate and high-risk chance of recurrence and progression^3^. Meta-analyses have demonstrated that BCG use can reduce recurrence by 28–56% compared with mitomycin C plus TUR or TUR-only treatments, though maintenance therapy is crucial to achieve these results^6,7^. Our data align with these findings, showing a 60% reduction in recurrence when BCG therapy was administered. When stratified by induction with or without maintenance, the lowest risk of recurrence was observed with BCG maintenance. Regrettably, only 13% of our patients received at least induction, and <3% of our patients received BCG maintenance, which may explain the nearly 50% recurrence rate with a median time of 27 months in our cohort. Typically, the expected recurrence rate for NMIBC is around 32–47%^4,11^, mostly during the first 2 years of follow-up, aligning with our observations.

Other variables associated with recurrence in our study were the presence of more than three lesions in the bladder and their size. These are well-known factors for recurrence, as previously demonstrated by Sylvester et al. in 2006^4^. Among these factors, and even after controlling for BCG use, the number of lesions identified at TURBT was independently associated with shorter RFS.

Bacillus Calmette-Guérin therapy can also reduce progression by 28%, which is a critical outcome because the progression to MIBC significantly impacts mortality^8^. However, this benefit seems to be limited to patients receiving maintenance therapy^8^. In our cohort, we did not observe any significant reduction in progression with BCG treatment. This could be attributed to the low use of BCG (13.9%) by our patients, particularly maintenance therapy (2.9%), and the low risk of progression (6.4%) during follow-up. Progression rates are expected to be around 11–13%, with follow-up around 46–69 months^4,11^. The shorter duration of follow-up in our study might explain the lower observed progression rate and the absence of a significant association with BCG use. However, we were able to confirm two important risk factors associated with progression: high grade and T1 stage. These are well-recognized prognostic factors for progression, as demonstrated by Sylvester in 2006^4^ and reaffirmed in 2021^5^.

Other groups worldwide have also demonstrated the impact of BCG shortage on the recurrence and progression rates with findings similar to ours. In South Korea, Lee et al. reported that BCG shortage and the presence of multiple tumors in the bladder were independently associated with recurrence in high-risk patients^12^. In 2023, Perez-Aizpurua et al. confirmed that reducing only a few doses of BCG led to increased recurrence rates of patients with high-risk disease^13^. Beyond clinical outcomes, the economic impact of the BCG shortage warrants attention. For instance, in Saudi Arabia, patients incurred costs of approximately EUR 1745 per patient^14^ due to the need to travel for the six-dose induction. In France, BCG shortage was associated with a higher recurrence rate and an increased cost of EUR 783 per patient^9^. Collectively, our findings and those from the literature highlight the importance of pursuing the BCG adjuvant treatment and might suggest that in the event of a BCG shortage, patients with high-risk disease should be prioritized for this treatment.

Our study has some limitations, including its retrospective nature, the lack of information about the number of doses of BCG received by each patient, and the low rate of patients receiving any form of adjuvant intravesical treatment. Despite these limitations, the study has notable strengths: it is the first to address this issue in a Brazilian cohort, all patients were treated according to consistent guidelines, and it includes a large patient population.

## CONCLUSION

Patients with intermediate- and high-risk NMIBC experience high recurrence rates, and the shortage of BCG might negatively impact these patients. If used according to guidelines, BCG could have reduced the recurrence rate by 60%.
